# Supplementary material for: Mapping Quantitative Trait Loci in Arabidopsis MAGIC Lines Uncovers Hormone-Responsive Genes Controlling Adventitious Root Development
Source: Plants (Basel). 2025 May 22;14(11):1574. doi: 10.3390/plants14111574 (PMC12157790; doi:10.3390/plants14111574)
Supplement: Supplementary file 1 [file plants-14-01574-s001.zip › Supplementary figures_S1_S2.pdf]

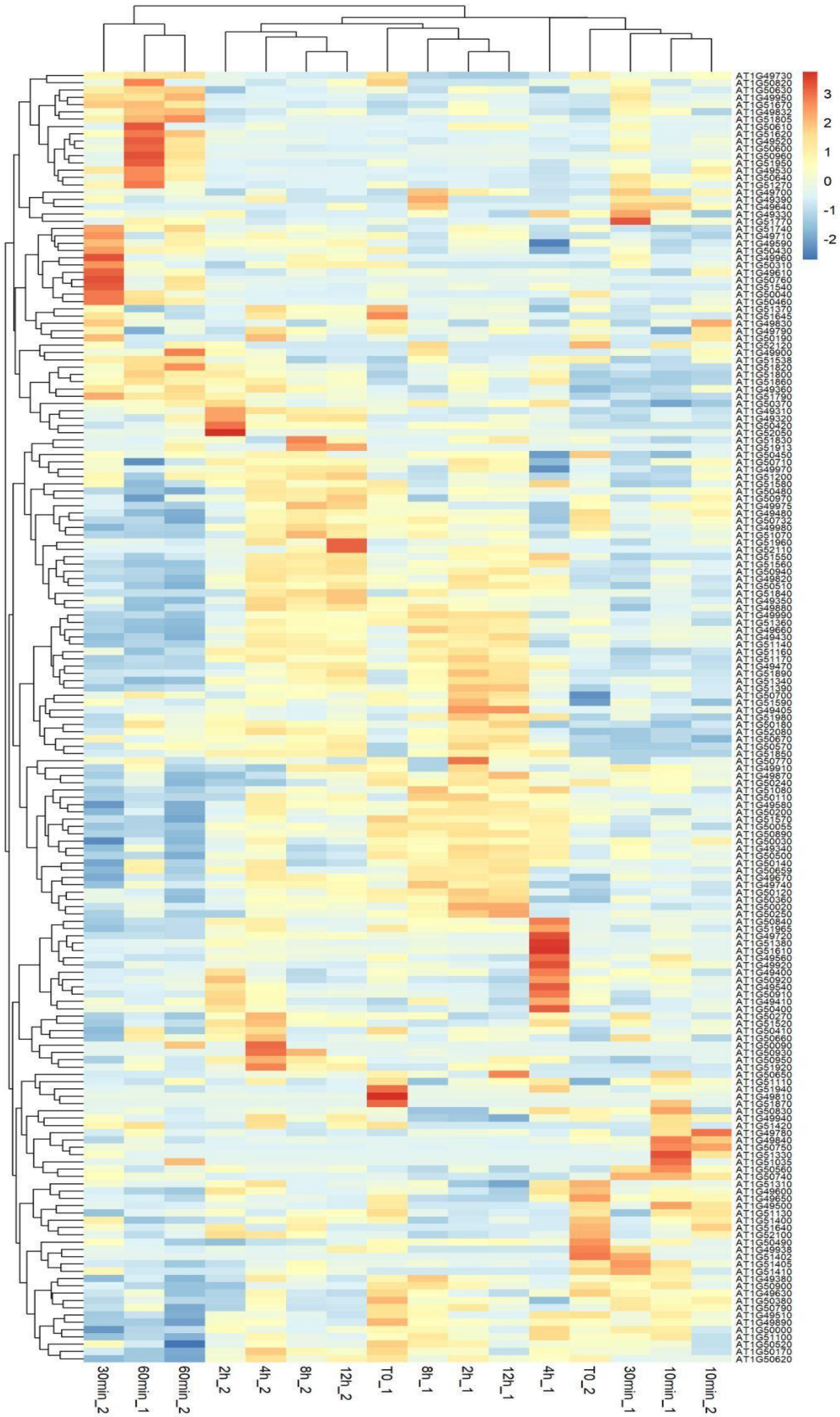

3
2
1
0
-1
-2
AT1G49730
AT1G50820
AT1G50630
AT1G49950
AT1G51670
AT1G49832
AT1G51805
AT1G50610
AT1G51620
AT1G49520
AT1G50600
AT1G50960
AT1G51950
AT1G49530
AT1G50640
AT1G51270
AT1G49700
AT1G49390
AT1G49640
AT1G49330
AT1G51770
AT1G51740
AT1G49710
AT1G49590
AT1G50430
AT1G49960
AT1G50310
AT1G49610
AT1G50760
AT1G51540
AT1G50040
AT1G50460
AT1G51370
AT1G51645
AT1G49830
AT1G49790
AT1G50190
AT1G52120
AT1G49900
AT1G51538
AT1G51820
AT1G51800
AT1G51860
AT1G49360
AT1G51790
AT1G50370
AT1G49310
AT1G49320
AT1G50420
AT1G52050
AT1G51830
AT1G51913
AT1G50450
AT1G50710
AT1G49970
AT1G51200
AT1G51580
AT1G50480
AT1G50970
AT1G49975
AT1G49480
AT1G50732
AT1G49980
AT1G51070
AT1G51960
AT1G52110
AT1G51550
AT1G51560
AT1G50940
AT1G49820
AT1G50510
AT1G51840
AT1G49350
AT1G49880
AT1G49990
AT1G51360
AT1G49660
AT1G49430
AT1G51140
AT1G51160
AT1G51170
AT1G49470
AT1G51890
AT1G51340
AT1G51390
AT1G50700
AT1G51590
AT1G49405
AT1G51980
AT1G50180
AT1G52080
AT1G50670
AT1G50570
AT1G51850
AT1G50770
AT1G49910
AT1G49870
AT1G50240
AT1G51080
AT1G50110
AT1G49580
AT1G50200
AT1G51570
AT1G50055
AT1G50890
AT1G50030
AT1G49340
AT1G50500
AT1G50140
AT1G50659
AT1G49670
AT1G49740
AT1G50120
AT1G50360
AT1G50020
AT1G50250
AT1G50840
AT1G51965
AT1G49720
AT1G51380
AT1G51610
AT1G49560
AT1G49920
AT1G49400
AT1G50920
AT1G49540
AT1G50910
AT1G49410
AT1G50400
AT1G50270
AT1G51520
AT1G50410
AT1G50660
AT1G50090
AT1G50930
AT1G50950
AT1G51920
AT1G50650
AT1G51110
AT1G51940
AT1G49810
AT1G51870
AT1G50830
AT1G49940
AT1G51420
AT1G49780
AT1G49840
AT1G50750
AT1G51330
AT1G51035
AT1G50560
AT1G50740
AT1G51310
AT1G49600
AT1G49650
AT1G49500
AT1G51130
AT1G51400
AT1G51640
AT1G52100
AT1G50490
AT1G49938
AT1G51402
AT1G51405
AT1G51410
AT1G49380
AT1G50900
AT1G49630
AT1G50380
AT1G50790
AT1G49510
AT1G49890
AT1G50000
AT1G51100
AT1G50520
AT1G50170
AT1G50620
30min_2
60min_1
60min_2
2h_2
4h_2
8h_2
12h_2
T0_1
8h_1
2h_1
12h_1
4h_1
T0_2
30min_1
10min_1
10min_2

Figure. S1. Heatmap of differentially expressed genes found in the QTL interval and common in a transcriptome of *de novo* adventitious root formation. We analyzed the available data from a time-lapse RNA-seq study of whole leaf explants from the Col-0 at various time points: t0, 10 minutes, 30 minutes, 1 hour, 2 hours, 4 hours, 8 hours, and 12 hours after leaf detachment using two replicates (_1 or _2) (Liu, 2022). We identified 177 of the 316 genes found in the QTL peak analysis, as shown in the heatmap. Genes that are downregulated are shown in blue, while those that are upregulated are indicated in red.

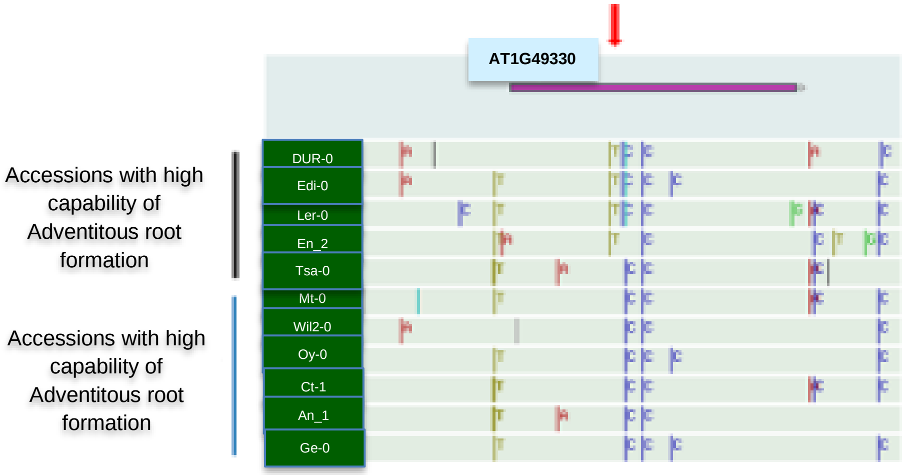

Figure S2. SNPs found in the gene that encodes for a Hydroxyproline-rich glycoprotein family protein in accessions described with variation in the number of AR formed after auxin treatment (King and Stimart, 1998). In the coding region, a missense variant (C/T; indicated with a red arrow) is observed in accessions with high AR formation (5.6 ± 0.6 to 7.2 ± 0.4 AR count), which is not present in accessions that produce a low number of AR (1.7 ± 0.3 to 3.8 ± 0.3). It is worth noting that En-2 and Ge-0 are not parental accessions used in MAGIC lines.
