# Supplementary material for: Mapping Quantitative Trait Loci in Arabidopsis MAGIC Lines Uncovers Hormone-Responsive Genes Controlling Adventitious Root Development
Source: Plants (Basel). 2025 May 22;14(11):1574. doi: 10.3390/plants14111574 (PMC12157790; doi:10.3390/plants14111574)
Supplement: Supplementary file 1 [file plants-14-01574-s001.zip › Table S10 Work-flow _QTL_analysis.pdf]

Supplemental Table S10. Step-by-step workflow for QTL mapping and gene overlay in Arabidopsis MAGIC lines

This table outlines the full computational workflow used to identify quantitative trait loci (QTL) associated with five root traits in a panel of 139 Arabidopsis MAGIC lines. The workflow was implemented in R using the `qtl2` and `atMAGIC` packages. Genotype probabilities were calculated from the pre-formatted MAGIC genotype object (`kover2009`) and combined with root phenotype data. A linear mixed model scan was conducted to identify QTL, and empirical genome-wide significance thresholds were derived via permutation testing (10,000 permutations per trait). Significant QTL peaks were extracted and plotted. For one trait (Num.RA), LOD support intervals were computed and overlaid with genome annotation data from the Arabidopsis BioMart database. This allowed us to identify all Arabidopsis genes located within the QTL interval, providing candidate genes for follow-up analysis.

The table includes the function(s) used at each step and explanatory notes, facilitating reproducibility and providing guidance for adapting the workflow to other traits or datasets.

Step	Description	R Function / Tool	Notes
1	Install and load necessary R packages	`install.packages()`, `library()`	Includes `devtools`, `qtl2helper`, `atMAGIC`, and Bioconductor gene annotation tools
2	Install and load genotype data	`data("kover2009")`	`kover2009` comes with the `atMAGIC` package
3	Read phenotype data file (`raiz(mean).txt`)	`read.table()`	Contains measurements for five root traits
4	Standardize subject IDs and remove extraneous columns	`gsub()`, subsetting by column	Ensures match with MAGIC line IDs in genotype data
5	Merge phenotype data with line label file (`HSRIL to MAGIC.csv`)	`merge()`	Maps internal sample IDs to official MAGIC line names
6	Attach phenotype data to genotype object	`add_pheno()`	Creates a unified data object for analysis
7	Calculate genotype probabilities	`calc_genoprob()`	Required for genome-wide QTL scan

8	Perform genome-wide QTL scan	`scan1()`	Performed for all five root traits simultaneously
9	Conduct permutation testing to determine empirical significance thresholds	`scan1perm(n_perm = 10000)`	10,000 permutations used per trait
10	Plot LOD profiles with trait-specific permutation thresholds	`plot(), abline()`	LOD scores visualized for each of the five root traits
11	Identify significant QTL peaks for a selected trait (e.g., Num.RA)	`find_peaks()`	Peaks above empirical threshold extracted and filtered by trait
12	Save significant QTL peaks to file	`write.csv()`	Outputs stored as CSV for downstream reporting
13	Determine LOD support interval for a selected peak	`lod_int()`	Computes genomic confidence interval for QTL region
14	Retrieve Arabidopsis gene coordinates from BioMart	`TxDb.Athaliana.BioMart.plantsmart12`	Provides reference gene annotations
15	Convert genes and LOD interval to GRanges for spatial overlap	`GRanges(), IRanges(), findOverlaps()`	Uses `GenomicRanges` and `IRanges` to identify genes within QTL interval
16	Save list of overlapping genes	`write.csv()`	List includes all genes located in the LOD interval
